# Effect of Physical Activity on Drug Expenditures for the Physical and Mental Health of Primary Care Users

**DOI:** 10.3390/ijerph23020221

**Published:** 2026-02-10

**Authors:** Diego de Melo Lima, Jamile Sanches Codogno, Glauciano Joaquim de Melo Júnior, Vilde Gomes de Menezes, Mariana Izabel Sena Barreto de Melo Cavalcanti, Eden Kaleo Soares da Silva, Flávio Renato Barros da Guarda

**Affiliations:** 1 Department of Physical Education, University of Pernambuco, Recife 50100-130, Brazil; diegomelo.lima@upe.br; 2 Department of Physical Education, Federal University of Pernambuco, Recife 50670-901, Brazil; 3 Faculty of Science and Technology, São Paulo State University Júlio de Mesquita Filho, Presidente Prudente 19060-900, Brazil; jamile.codogno@unesp.br; 4 Department of Nursing, Federal University of Pernambuco, Recife 50670-901, Brazil; 5 Department of Public Heath, Federal University of Pernambuco, Vitória de Santo Antão 55608-680, Brazil

**Keywords:** primary healthcare, health economics and organizations, physical activity, Unified Health System (SUS)

## Abstract

**Highlights:**

**Public health relevance—How does this work relate to a public health issue?**

- Insufficient physical activity is associated with higher medication use and expenditures in primary health care.
- Psychotropic drug use represents a substantial share of public spending on mental health within the Unified Health System (SUS).

**Public health significance—Why is this work of significance to public health?**

- Physically active primary care users showed lower public spending on overall medications and psychotropic drugs.
- Habitual physical activity emerged as a potentially protective behavior against increased pharmaceutical expenditures.

**Public health implications—What are the key implications or messages for practitioners, policy makers and/or researchers in public health?**

- Physical activity promotion strategies may contribute to reducing public spending on medications.
- Health policies that encourage active lifestyles may strengthen the sustainability of public health systems.

**Abstract:**

The primary and secondary objectives of this article are, respectively, to measure the effect of habitual physical activity on total medication expenditures and on expenditures specifically related to psychotropic drugs among primary healthcare users in a large Brazilian city. This cross-sectional study with a retrospective component was conducted using Propensity Score Matching (PSM). PSM is a robust and widely utilized method in studies evaluating the impact of public policies, particularly in observational data settings where randomization is infeasible. Medication expenditures and habitual physical activity data referring to the past 12 months were collected from 250 users of both sexes, aged over 40 years, across seven primary healthcare units. The average medication expenditure was USD 6.33 (95% CI: −206.64 to −31.02), and for psychotropics, USD 0.63 (95% CI: −217.75 to −11.87). The effect of physical activity on expenditures showed that more active individuals spent on average USD 34.83 less on all medications and USD 4.34 less on psychotropics compared to less active individuals. The findings of this study reinforce the importance of the physical activity as a health promotion strategy and as a means to reduce public health expenditures.

## 1. Introduction

Medicines are technologies that place the greatest burden on healthcare systems [1]. Spending on pharmaceutical assistance was responsible for up to 60% of the health budget in developing and middle-income countries at the beginning of the 21st century [1], and has been increasing in recent years [2].

In 2015, per capita spending on medicines in general in primary health care was lower in countries with universal systems (USD 77.00) than in those with social insurance systems (USD 99.00) [3]. Spending on medicines in the Brazilian Unified Health System in 2016 was BRL 18.6 billion, which represented an increase of 30% compared to the previous five years [4].

Among the spending on medicines, spending on psychotropic medicines stands out, having increased globally [5,6,7], mainly due to improved diagnosis of mental disorders [8] and increases in the incidence of anxiety [9] and depression [10].

Spending on psychotropic medicines is responsible for an important portion of health spending [2]. In 2020, medicines aimed at mental health were responsible for spending of approximately USD 32 billion worldwide [11]. In the United States of America, psychotropic medicines generated an spending of USD 57.78 million in 2012, with a projected increase to USD 71.56 million in 2020, representing, respectively, 21% and 17% of spending on all medicines [7].

Evidence shows that in Brazil, overall spending on medicines was approximately USD 16.1 billion between April 2007 and March 2014. Furthermore, spending on medicines for the central nervous system, including psychotropic medicines—which are sold exclusively upon presentation of a medical prescription issued in two copies—increased by 150% between 2006 and 2012, reaching USD 54.6 million, which positions them as the fifth class of medicines that most burdened the Unified Health System (SUS) in the last decade [2].

Psychological care and the use of psychotropic medicines are the two main recommendations for the treatment of common mental disorders [12]. However, the regular practice of physical activities is an important strategy for improving the physical and mental health of the population [13,14].

Research shows that regular physical activity enhances quality of life and alleviates symptoms for individuals with mental disorders [13]. Additionally, maintaining an active lifestyle is linked to reduced healthcare costs, lower overall medication use, and decreased reliance on psychotropic medications [15,16,17,18,19,20,21].

Encouraging the regular practice of physical activity (PA) is part of the set of actions aimed at improving the mental health of patients of Primary Health Care in Brazil, and forms part of the scope of the National Mental Health Policy [22]. In this sense, evaluating the impact of physical activity on public spending on medicines is an important strategy to support decision-making processes in the management of health services, and to support the implementation of public policies informed by evidence.

The city examined in this research has the fourth-largest population among the 185 municipalities in the state of Pernambuco, totaling 369,343 residents. It also ranks third in state spending on health actions and services, with an expenditure of USD 38,612,887.14. The city’s health department implements various actions and programs aimed at promoting physical activity as a primary health care strategy to enhance both the physical and mental well-being of its residents [23,24].

In 2019, the expenditure on medicines reached USD 8,093,958,225, representing 4.58% of total pharmaceutical assistance spending across the state and accounting for 0.58% of all municipal public spending that year [25].

Some evidence in the literature indicates that regular physical activity is able to prevent diseases [26], improve physical and mental health [13,14], and mitigate public spending on health actions and services [16,17,18]. Furthermore, PA reduces the incidence and severity of chronic conditions, endothelial function, and insulin sensitivity, thereby decreasing the need for ongoing pharmacotherapy and hospitalizations, mitigates mental health disorders like anxiety and depression—prevalent drivers of psychotropic drug use—via enhanced neurotransmitter regulation (e.g., serotonin, BDNF) and reduces inflammation, leading to fewer outpatient visits and medication prescriptions [16]. On the other hand, the relationship between the practice of physical activities by primary care patients and public spending on medicines is not yet clear.

Therefore, the primary objective of this study was to measure the effect of habitual physical activity (HPA) on total public spending on medicines, while the secondary objective was to evaluate the effect of this behavior on specific spending on psychotropic medicines in patients of Primary Health Care in a large city in Northeastern Brazil.

## 2. Materials and Methods

This is a cross-sectional study with a retrospective component, conducted using an econometric approach based on Propensity Score Matching (PSM) (PSM) [27].

The research was carried in a large city in Northeastern Brazil. The city’s population is 365,278 inhabitants, with a per capita Gross Domestic Product of BRL 20,028.26 (or USD 3859.01; 12th in the state and 2396th among Brazil’s 5570 municipalities), an average income for formal workers of 1.7 minimum wages, and a high Human Development Index of 0.721 (20th highest in the state and 1800th among Brazil’s 5570 municipalities) [28].

The city’s health network is made up of 73 Basic Health Units (BHU) that serve 69% of the population, in addition to two Psychosocial Care Centers (CAPS), four centers of the national health and physical activity promotion program (Health Gyms), 12 multidisciplinary teams to support the actions of primary health care teams (NASF-AB), 27 specialized clinics/outpatient clinics, and seven hospitals [29].

For the current research, seven Basic Health Units (BHU) were selected considering the following criteria: (i) covering urban and rural areas; (ii) have a Medical Electronic Citizen Record implemented for at least one year; and (iii) be served by multidisciplinary teams to support primary health care actions. The selection of these seven BHU also took into account their geographic distribution (north, south, central-east, and west) and population size (number of inhabitants and available health services).

Patients of both sexes were selected through an invitation extended in the BHU waiting room. Previously trained researchers explained the research procedures and asked participating users to sign an Informed Consent Form. A post hoc power and precision assessment was conducted to evaluate the adequacy of the available sample size, using the StatCalc module available in the web-based version of Epi Info™ (Centers for Disease Control and Prevention—CDC), based on the observed prevalence of general medication use (49.1%) [30] The parameters included a 95% significance level (α = 0.05), an acceptable margin of error of 10% (E = 0.1), and a design effect of 2.0, ensuring sufficient precision for prevalence estimates [31]. Additionally, a post hoc power analysis using G*Power (3.1.9.7) confirmed statistical power above 80% to detect moderate associations (e.g., Cohen’s d = 0.5) between habitual physical activity and expenditure differences at α = 0.05, supporting the robustness of the PSM analyses despite the fixed sample derived from available records.

**Figure 1:**
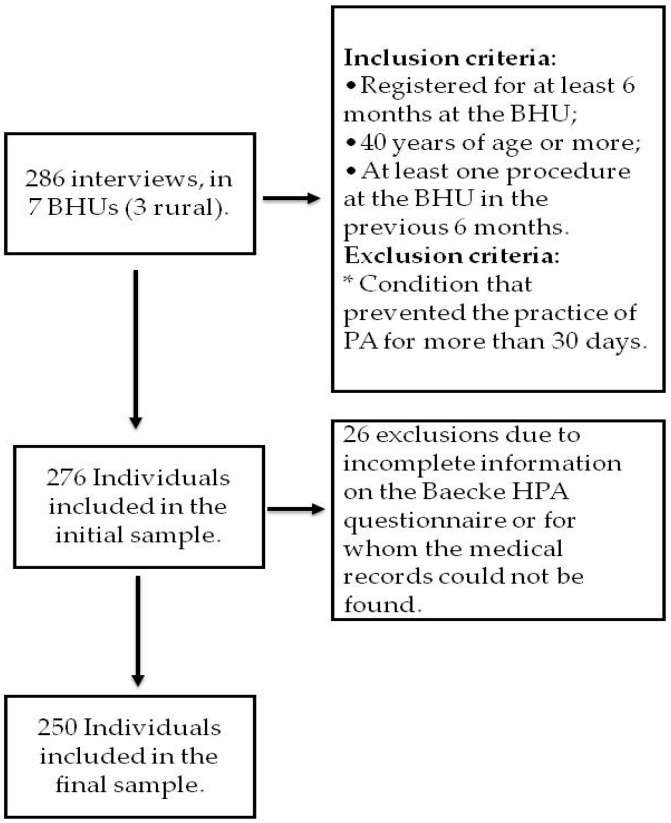
Sample selection flowchart and inclusion and exclusion criteria.

Based on this precision assessment, the estimated number of individuals required to evaluate general medication use was 221, considering an initial available sample of 192 patients and an additional 15% to account for potential losses related to incomplete medical records or missing questionnaire responses. The sampling process, losses, and inclusion and exclusion criteria are shown in Figure 1.

The primary data (sociodemographic, economic characteristics, and those related to the practice of Habitual Physical Activity) were collected from December 2019 to March 2020.

Secondary data (quantity and type of medications prescribed) were removed from the Citizen’s Electronic Health Record, with the express consent of the City Health Department, and ensuring the anonymity of the patients. In order to avoid sample loss in cases where the electronic medical record was not found, a new visit to the health units was carried out to access the physical records.

Spending on medicines (total and specific to psychotropic medicines) was calculated based on the values contained in the health price database (BPS) for the month of February 2020 (106 medications). For medicines whose values are not included in the BPS, consultations were carried out with the Price Panel of the Ministry of Economy. For medications whose prices were not available in the BPS, data were sourced from the Price Panel of the Ministry of Economy (one medication). When this information was unavailable, market prices were adopted (two medications), calculated as the average of the values obtained from three major regional pharmacies.

The primary (total spending on medicines) and secondary (specific spending on psychotropic medicines) dependent variables were calculated by multiplying the price by the number of each medicine prescribed in the Citizen’s Electronic Health Record in the year prior to the date of collection for each individual.

Brazil adheres to international clinical protocols while implementing its own national guidelines for psychotropic medication prescribing, primarily through the Ministry of Health’s Clinical Protocols and Therapeutic Guidelines (PCDT), the Guidelines of the National Mental Health Policy, and the Guides for Rational Use of Medications. These frameworks mandate that prescriptions be grounded in accurate clinical diagnosis, prioritize non-pharmacological interventions whenever feasible, and promote rational and safe psychotropic use to minimize risks such as dependency and adverse events [2,32].

This study considered the following medicines to be psychotropic: alprazolam, carbamazepine, lithium carbonate, clomipramine, clonazepam, amitriptyline hydrochloride, biperiden hydrochloride, clomipramine hydrochloride, chlorpromazine hydrochloride, fluoxetine hydrochloride, nortriptyline hydrochloride, diazepam, phenytoin sodium, phenobarbital, phenobarbital sodium, fluvoxamine, haloperidol, imipramine, biperiden lactate, moclobemide, paroxetine, sertraline, magnesium sulfate, sodium valproate or acid, valproic acid, and venlafaxine [32,33].

After calculating all expenses in Reais (Brazilian Currency), inflationary correction of the values was carried out based on the National Consumer Price Index accumulated between the months of February 2020 and August 2021, followed by conversion to the American Dollar, using the price on 30 August 2021 (USD 1.00–BRL 5.19).

The assessment of habitual physical activity (HPA) in the previous year was carried out using the Baecke questionnaire, which is characterized as a recall of physical activity in the last 12 months. This instrument was validated in Brazil and presents good psychometric characteristics [34].

The Baecke recall generates a score, which is the sum of the score for the practice of occupational and sports activities during the leisure period and the practice of leisure and locomotion activities. This inventory considers that the higher the value obtained by an individual, the greater the practice of PA in the previous year. In this way, individuals were classified as “more active” (Baeke score values from the 75th percentile) and “less active” (scores below the 75th percentile). A study using accelerometers found that individuals classified above the 75th percentile can be considered sufficiently active (≥150 min per week of moderate aerobic activity or an equivalent amount of vigorous activity—MVPA). Validation tests indicated a sensitivity of 59–77% and specificity of 71–89% for identifying higher levels of MVPA (accelerometer cutoff: 2020+ counts/min), which aligns with World Health Organization criteria for sufficient activity [35].

For the current study, the more active individuals were designated as treated and the less active individuals were considered as controls in the econometric analyses.

The variables that made up the estimation model were chosen considering the evidence in the literature that points to the socioeconomic, demographic, and epidemiological factors associated with spending on medicines in the Brazilian population [19,36,37].

The descriptive statistics of the numerical variables are presented as the mean, standard deviation, and confidence interval at the 95% level (95%CI) and stratified between the “More Active” and “Less Active” groups. Categorical variables are presented in absolute and relative frequency.

The econometric analyses were carried out according to the following steps: (i) estimation of the HPA effect through propensity score matching; (ii) estimation of a reweighting model using the inverse of the propensity score; (iii) calculation of a doubly robust estimator to assess the effect of HPA on spending; and (iv) testing compliance with PSM assumptions.

It is noteworthy that the main method for evaluating the effect of HPA on expenses (total and with psychotropic medicines) used was Propensity Score Matching, which aims to minimize problems related to the non-randomness of sample selection [38,39]. The other methods were used as alternatives to compare the findings and to attest to the robustness of the results found [40,41].

### 2.1. Estimation of the Effect of HPA Through Propensity Score Matching

In order to overcome the problem of selection bias, we chose to use a strategy that allows comparison between the more active and less active individuals through propensity score matching (PSM). This method makes it possible to find, in the control group (“Less Active”), individuals with observable characteristics (such as sex, skin color, age group, and level of education) similar to those of the individuals in the treated group (“More Active”) by calculating the conditional probability of an individual being treated [27].

PSM produces a good estimate of the average treatment effect on the treated (ATT), provided that the assumptions of conditional independence or ignorability are met, as well as the assumption of overlap or common support [27]. In this study, propensity score matching was calculated using the “nearest neighbor” algorithms (1:1 and 1:5, both with replacement), Radial and Kernel Matching [42].

### 2.2. Reweighting by the Inverse of the Propensity Score

The use of the inverse of the propensity score (Inverse Probability Weighting—IPW) as a weight in a regression aims to remove the influence associated with the fact that the individual is observed in only one of the following situations: more active (treated) or less active (control). In other words, the estimator minimizes selection bias by ensuring that treatment assignment is random [38]. To do this, the estimator assigns greater weights to treated individuals who have the lowest probability of being more active [38,39].

### 2.3. Calculation of the IPWRA Doubly Robust Estimator

Adjusted regression weighted by the inverse of the propensity score (Inverse Probability Weighted Regression Adjustment—IPWRA) was also used as an alternative to PSM. This estimator considers two models, one for the variable of interest, and the other to calculate the probability of treatment, using the weights corresponding to the inverse of the treatment probabilities in the adjusted regression of the variable of interest. This estimator is considered doubly robust for impact assessment and is consistent even when only one of the models is correctly specified [40].

### 2.4. Compliance with PSM Assumptions and Quality

Two tests were performed to verify the quality of the matching. Verification of compliance with the common support assumption was carried out by comparing the means of the observable variables of the “More Active” and “Less Active” groups before and after matching [43], with results presented through a graph and a table.

Three placebo regressions were used to indicate whether the ignorability hypothesis could be taken as true. To this end, all explanatory variables utilized in estimating the propensity score were used, but with different dependent variables (spending on dental appointments, and Body Mass Index), which we assume to be exogenous to the treatment. In the event that there is an omitted variable related to the treatment, it is expected that the estimated coefficients for being “more active” are statistically different from zero. On the other hand, if the coefficients of the placebo regressions are statistically equal to zero, it can be said that the ignorability condition has been met [41] and that the matching model is efficient to evaluate the effect of HPA on medicine spending.

## 3. Results

The mean age of the sample was 56.48 years (9.88 and 95% CI 55.25–57.71), and female individuals predominated (78.4%), attended in urban BHUs (82.8%), who did not work (no job, no income, or retired) (64.4%), had not completed elementary education (67.6%), and belonged to the middle class (class C) (49.6%). Regarding mental health, it was observed that the diagnosis of depression was more prevalent (*n* = 21; 8.4%) than that of anxiety (*n* = 17; 6.8%).

The mean HPA score was 4.617 (SD ± 1.142; median 4.5), with those from the 75th percentile being considered more active (*n* = 53; 21.2%), that is, individuals whose HPA scores were greater than 5.25.

The “more active” group included male individuals with some type of employment (formal or informal) and with higher education (completed higher education). Table 1 presents in detail the characteristics of the more and less active individuals in each of the categorical variables analyzed.

In the total sample, 149 individuals (59.6%) used some type of medicine and 30 (12%) used psychotropic medicines. The total expenditure on medications over the study period was USD 2085.25, and spending on psychotropic drugs amounted to USD 207.23. The mean total spending on medicines was USD 6.33 (SD + 30.96; Median USD 0.74) and specific spending on psychotropic medicines was USD 0.63 (SD + 4.37; Median USD 0.00).

Table 2 presents the descriptive statistics for total spending on medicines and specific spending on psychotropic medicines among the more active and less active individuals.

Spending on medicines was significantly higher among those diagnosed with depression (mean USD 34.07; Median USD 4.76; Difference = USD 30.29; SD 100.064; *p* = 0.000). Regarding spending on psychotropic medicines, a significant difference was observed in spending for the skin color variable, in which white individuals had higher mean expenses (mean USD 1.25; Median USD 0.00; Difference = USD 1.05; SD 6.728; *p* = 0.031), as did individuals diagnosed with depression (mean USD 5.93; Median USD 2.16; Difference = USD 5.79; SD 13.828; *p* = 0.000).

### 3.1. Effect of HPA on Spending on Medicines in General and on Psychotropic Medicines

The variables used in the model to evaluate the effect of habitual physical activity on total spending on medicines and specific spending on psychotropic medicines were as follows: the BHU location area (urban or rural), sex, age group, occupation, race/color, level of schooling, socioeconomic classification, and diagnosis of depression.

The results describe the estimations carried out through propensity score matching and for the IPW and IPWRA estimators.

The estimation through Propensity Score Matching using the nearest neighbor algorithm (N1) was a more adjusted regression and demonstrated the best effects of HPA on expenses. The results indicate that in the previous 12 months, the more active individuals spent an average of USD 34.83 (standard error + 12.177) less on medicines and USD 4.34 (standard error + 1.698) less on psychotropic medicines than the less active individuals.

The other estimators used also indicated a significant reduction in spending on medicines and psychotropic medicines. Table 3 presents the coefficients of the estimators that evaluated the difference in spending on medicines between “more active” and “less active” individuals and the statistical significance of the tests.

### 3.2. Compliance with PSM Assumptions and Quality

**Figure 2:**
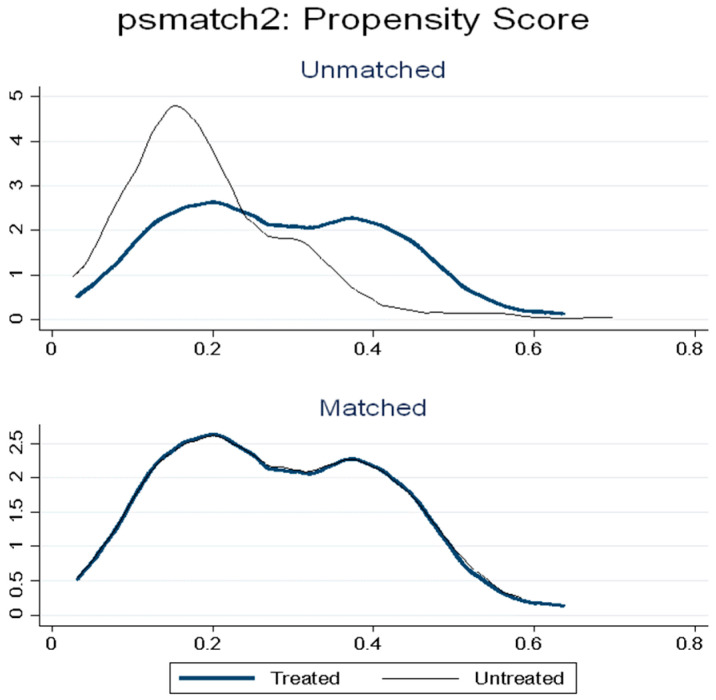
Distribution of treatment probability before and after matching. **Source:** produced by the authors.

The graphical analysis of the probability distribution presented in Figure 2 shows that the distribution of the values of the vector of observable characteristics of the more and less active individuals was different before matching, with the less active being concentrated in the left tail of the distribution. After matching (one nearest neighbor with replacement) the area of common support between treated and controls (in the center of the distribution) was expanded, indicating good matching quality.

Table 4 presents the means of the variables for the control and treated groups before and after matching and allows checking the balancing conditions in the treatment distribution. The result of the mean comparison test allows us to state that after matching the groups became statistically equal (and only different in relation to HPA).

The three placebo regressions, carried out in order to verify whether the PSM model meets the hypothesis of ignorability, presented non-significant coefficients for the dependent variables tested (BMI and Spending on Dental Consultations), indicating that the model adopted is valid and contains the information necessary to point out the potential result in the absence of treatment in terms of total spending on medicines and spending on psychotropic medicines. Table 5 presents the results of the placebo regressions.

It is worth noting that as the models for evaluating the effect of HPA on total spending on medicines used the same covariates, it was not necessary to test different placebo regressions to evaluate the effect on total spending and on specific spending on psychotropic medicines.

## 4. Discussion

The sample evaluated in the current study showed a prevalence of 8.4% for depression and 6.8% for anxiety. These values are above the national average for the prevalence of depression and below the national average for anxiety, respectively, 5.8% and 9.3%, according to surveys carried out by the World Health Organization [44]. However, the prevalence of depression was lower in city studied than in the sample of Brazilian adults using BHU from all over Brazil (18.5%) and the northeast (15.2%) [45]. The prevalence of anxiety was also lower than that observed in older people in a capital city in another state of northeastern Brazil (48%) [46].

In the present study, the HPA score was 4.617 (median 4.5 points) in the total Baecke recall. Codogno et al. [36], in a study also carried out with BHU patients in a municipality in the interior of the state of São Paulo (Southeastern), observed higher scores for leisure and locomotion activities (median 3.00 in both men and women) when compared to the practice of sports activities (median men 1.16; and median women 1.00).

The adoption of an active lifestyle is related to several social, epidemiological, and behavioral factors [47]. Our finds indicated that men (22.2%) with higher education (36.4%) are more active. These results are similar to a study carried out with Brazilian adults in which active men with higher education represented 32.7% and 39.2% of the sample, respectively [48]. However, Silva and colleagues [48] observed a higher prevalence of physical activity among white individuals (31.9%), which was not found in our study, in which white individuals represented only 15.5% of the “more active” individuals.

The mean total spending on medicines per person in this study (USD 6.33) was lower than the mean observed in another city in São Paulo state (USD 26.92) [19], and in other countries with universal healthcare systems (USD 77.00) [3]. The differences found between the studies may be related to the method used to calculate medicine costs. While the current study used data from the health price database, the study by Fernandes et al. [49] used the Brazilian Public Health System table or the market price when the medicines were purchased by research participants, and the study by Morgan et al. [3] used data from an international price database that considers the market prices of medicines.

The significant variation in medication expenditure values observed in our study, as indicated by the high standard deviations for both general and psychotropic drugs, reflects a well-established pattern in Brazilian primary health care (PHC) within the Unified Health System (SUS). This variation is driven by patient-specific factors, including differences in the prevalence of chronic diseases, the burden of multimorbidity, and high rates of anxiety and depression. These factors contribute to skewed distributions that often include frequent zero values (representing the absence of diagnosis or discontinuation of treatment for underlying diseases and their respective comorbidities) alongside outliers resulting from intensive psychotropic regimens for severe cases.

Vieira and colleagues [50] reported that 25% to 45% of municipal PHC datasets showed zero expenditures due to discrepancies in capitation rates and recording inconsistencies. Meanwhile, Rossignoli et al. [51] highlighted intra-municipal price fluctuations for essential drugs, ranging from 32% to 482%, which further increased variability due to differences in procurement practices. Additionally, Turi et al. [52] documented that top-decile patients accounted for 30% to 50% of outpatient costs in PHC cohorts, emphasizing how individual clinical needs can distort expenditure profiles.

These structural and epidemiological factors, together with high-cost claims resulting from judicial demands (which can account for up to 33% of budgets), provide context for our findings. Thus, our results are representative of the realities faced in SUS PHC, rather than being methodological artifacts.

Medicines for the central nervous system were the fifth class of medicines that most burdened the public health system in Brazil in 2014 (USD 54.6 million), representing 1.9% of medicine spending in the country [2]. In this sense, although spending on psychotropic medicines has increased in recent years [2,5,7], in our results, we observed spending more than five times greater (10% of spending on medicines) than that observed throughout Brazil. The greater spending on psychotropic medicines may be related to the population assessed, which was made up of active BHU patients, many of whom routinely go to the units to collect their medicines for continuous use [53].

A study carried out in Brazil indicated that the majority of psychotropic medicine users routinely visit health units, among whom most do not have a mental disorder that would justify the use of these medicines [53]. However, in the present study, patients with depression had higher expenses for both medicines in general (+USD 30.29) and psychotropic medicines (+USD 5.79). Studies that deal with the expenses generated by patients with depression, summarized in a systematic review, showed the highest expenses incurred by these individuals in several variables related to health expenses [37].

Loch and colleagues [54] showed leisure-time PA reduced depressive symptoms odds by 26% (OR = 0.74), particularly at 121–360 min/week, mediating reduced psychotropic prescriptions amid domain-specific activity patterns. Furthermore, a 2023 systematic review confirmed PA causally lowers incident depression/anxiety (strong inverse associations), estimating population-level PA increases could avert cases needing pharmacotherapy, with mental health cost savings modeled via reduced disorder burden [55].

It is noteworthy that the amount spent on psychotropic medicines increased 2.5 times between 2010 and 2015 [5].

The results of this the sample in the current study showed that white individuals generate greater spending on psychotropic medicines (+USD 1.05; *p* < 0.05). This finding reinforces the results of a study carried out in another similar city in southeastern Brazil, in which the prevalence of psychotropic medicine use was higher in white individuals [56]. The higher spending among white individuals aligns with well-documented racial inequalities in healthcare access, where white populations exhibit greater utilization of specialized services, diagnostics, and medications due to better geographic proximity to facilities, higher socioeconomic status, and reduced institutional barriers compared to Black individuals [57].

Regarding the econometric modeling used in this study, the variables that made up the PSM model (main analysis) and the alternative models (IPW and IPWRA) reiterate the evidence from the literature that indicates that sex, age group [58], occupation [59], race/color [60], level of schooling [61], and socioeconomic classification [47] are associated with the level of physical activity, and that this behavior is associated with lower spending on medicines [16,19,52].

Propensity Score Matching (PSM) proves particularly appropriate for evaluating the causal impact of habitual physical activity levels on medication spending, as it addresses selection bias inherent in observational questionnaire and electronic health record data where more active individuals systematically differ from less active ones across observables like age, BMI, and socioeconomic status. Unlike correlation-based studies that merely document associations (e.g., negative bivariate correlations between activity scores and psychotropic expenditures), PSM constructs a counterfactual by matching treated (“more active”) individuals to untreated controls with similar propensity scores, effectively balancing covariates and isolating the average treatment effect on the treated (ATT) [62,63,64,65,66]. This quasi-experimental approach yields robust causal estimates, whereas correlation analyses risk overestimating effects due to confounding (e.g., healthier socioeconomic profiles driving both activity and lower spending). By leveraging anthropometric measures and clinical records for comprehensive covariate adjustment, PSM satisfies ignorability conditional on observables, providing stronger internal validity than simple regressions for policy-relevant inferences on activity-driven pharmacoeconomic savings [27,41,42].

All methods used to estimate the effects of HPA on total spending on medicines and specific spending on psychotropic medicines indicated that more active individuals generated lower spending than less active individuals. This increases the comparability between estimates [39,62,63].

In the current study, BHU patients classified as “less active” could have different characteristics from those present in “more active” users, due to the heterogeneity that may be present in the observations [64]. In this case, Propensity Score Matching is an alternative to minimize potential selection bias, as it allows the formation of groups of more active and less active users that are similar in their observable characteristics (therefore comparable) and different only in relation to the fact that they are more or less active [63,65].

The statistically significant results obtained by the IPW and IPWRA estimators guarantee consistency in the models used to verify the effect of habitual physical activity on expenses [40,66]. The first estimator models the possibility of being “more active” to explain the non-random assignment to this condition. Additionally, the IPWRA estimator, in addition to modeling the treated group (being more active), also makes assumptions regarding the variable of interest (total spending on medicines and specific spending on psychotropic medicines) [39].

The IPW and IPWRA estimators are part of a set of methods that aim to measure treatment effects that refer to the average causal effect (Average Treatment Effect—ATE) that binary variables exert on outcome variables present in policy contexts or a behavior [39], as is the case of HPA in this study, controlling the results for certain characteristics inherent to BHU patients.

It was not possible to compare the effect of HPA on total spending on medicines and psychotropic medicines found in this study and with other research, as no studies with similar methodology were found. However, our results reinforce what other studies have already shown—namely, that physical activity can reduce health costs [67] and hospital admissions [68], and that active individuals use less medicines [16].

The difference in spending on medicines and psychotropic medicines between more active and less active individuals, after matching, may be related to the effects of physical activity on some risk factors for chronic diseases, which lead to a reduction in the risk of developing these diseases [26] and has been observed in several studies [17,19,69]. Evidence shows that practicing physical activity is able to reduce overweight and obesity [70], control metabolic rates, such as triglycerides and blood glucose [71], reduce and maintain blood pressure at healthy levels [72], and stimulate the release of hormones that promote the reduction in symptoms of anxiety and depression [73].

The three analyses carried out to evaluate the quality of the model matching reinforce the validity and reliability of the results. In the first, the graphical analysis demonstrated compliance with the common support assumption of Propensity Score Matching, that is, it was possible to affirm that BHU patients with the same propensity score had a positive (and similar) probability of being more active (treated) or less active [74], because although the distribution of the values of the vector of observable characteristics was slightly different before matching, this method minimized these differences and form comparable groups [43].

Also noteworthy is the assessment of the quality of the matching, which was confirmed by comparing the average characteristics of the more active and less active users. This procedure reinforces that after matching for all covariates, it was not possible to reject the null hypothesis of equality of means and, therefore, there is matching with a good balance between the observable characteristics of the “more active” and “less active” individuals [43,64].

The last robustness test of the results of this study consisted of carrying out placebo regressions, which sought to verify the assumption of ignorability of the PSM [41], that is, whether the vector of observable characteristics used in the regression models contained all variables that potentially affect spending on medicines in the absence of treatment (when individuals are less active). In the three placebo regressions tested, the estimated coefficient for being “more active” was statistically equal to zero, which indicates that there is no omitted variable problem and that the ignorability hypothesis can be accepted as true [41].

Considering that population-based studies are one of the best ways to collect information about the health of the population [75], it can be suggested that the main limitation of this study is the fact that the indicated units did not serve all territories or regions of the analyzed city, so that some particularities of the territory may have been disregarded. However, these units met the criteria established for the research, and the analysis method used (PSM) minimizes the problem of non-randomness, as it compares individuals with the most similar characteristics [65].

We also acknowledge the following limitations: (1) the use of non-random (convenience) sampling from the available database, which may limit representativeness; (2) potential selection bias due to reliance on health service users, who may differ from the general population in healthcare-seeking behaviors; and (3) restricted external validity, as findings may not generalize to other municipalities or socioeconomic contexts beyond our setting.

Notably, the primary data analysis method—propensity score matching (PSM)—helps mitigate the lack of randomization by balancing observed covariates between groups, reducing confounding and selection bias to approximate a quasi-experimental design, thereby enhancing internal validity despite the non-random sampling.

This transparent discussion strengthens the manuscript by contextualizing these inherent limitations of cross-sectional designs using secondary data, consistent with recent methodological reviews [76].

The use of a quasi-experimental methodology is one of the innovations of this study. PSM is commonly utilized in evaluation studies, especially on the impact of public policies [42,60], however, it is little explored when evaluating the effect of a behavior, as is the case of HPA. It is important to emphasize that while PSM is a robust approach, it cannot fully eliminate the possibility of unmeasured confounding variables.

We also agree that recall bias is a potential limitation regarding physical activity practice in the last 12 months. Nonetheless, it is worth noting that this behavior was assessed with the Baecke Habitual Physical Activity Questionnaire, a widely used tool specifically designed to capture habitual activity over the previous 12 months. Validation studies in Brazilian adults have demonstrated acceptable reliability (intraclass correlation coefficients between 0.69 and 0.80) and concurrent validity for the past-year recall period, supporting its use in epidemiological research [34,35]. More recently, Tebar et al. [35] showed that the Baecke questionnaire presents good reliability and moderate validity when compared with accelerometer-measured physical activity, reinforcing its suitability as a measure of habitual physical activity over the preceding year.

Finally, even though the selection of the BHU has taken into account their geographic distribution (north, south, central-east, and west) and population size (number of inhabitants and available health services), some patients may systematically differ from non-attendees in ways affecting both physical activity and medication use.

## 5. Conclusions

The current study aimed to measure the effect of HPA on total spending on medicines and specific spending on psychotropic medicines. The analyses carried out allow us to affirm that more active BHU patients generate lower public spending on measures for general use and psychotropic medicines.

In this study only 21.2% of the individuals studied were classified as more active, and they spent, on average, USD 4.34 less than the less active individuals. This corresponds to an approximate annual saving of USD 230. In this sense, for every 20% increase in the proportion of more active individuals, it is possible to save approximately USD 47.80 per year. If at least 75% of individuals were classified as more active, the savings on overall medication expenditures would reach USD 811.50, representing 1.89% of the total pharmaceutical care spending in the municipality for the year 2019.

The results of this research reinforce the importance of practicing HPA for the health of the population and for reducing public spending, whether on medicines in general or psychotropic medicines. Therefore, it is inferred that increasing the practice of HPA in the population can reduce spending on medicines in general, as well as on psychotropic medicines.

The use of PSM, a quasi-experimental method widely used in econometric studies, enabled comparisons to be made between the more and less active groups, eliminating possible biases related to sample selection, and the model used proved to be robust in all analyses performed. In this way, the methodological design used can serve as a basis for other studies that evaluate the effects of physical activity on health-related outcomes and encourage the development of public policies informed by evidence. On the other hand, we highlight that more robust causal evidence—such as longitudinal cohort studies or intervention trials—will be necessary before these cost estimates can be used directly in formal economic modeling or for decision-making in health technology assessment.

The placebo tests conducted resulted in coefficients statistically equal to zero. This finding indicates that only general medication spending and psychotropic spending are influenced by habitual physical activity, and that the PSM model estimated for this study is sufficiently robust to assess the impact of this behavior. Failed placebo tests, however, would imply systematic omitted variable bias, rejecting ignorability and invalidating PSM-based estimates, as unmeasured confounders would spuriously correlate with treatment assignment and psychotropic spending outcomes.

Finally, this study reinforces the importance of regular physical activity as a strategy for promoting physical and mental health within Brazil’s Unified Health System (SUS), through both public exercise programs—such as the Academia da Saúde initiative and multidisciplinary teams with physical educators supporting primary care units—and incentives for autonomous leisure or commuting-based activity, per the Ministry of Health’s physical activity guidelines. Our findings indicate that increasing population activity levels—particularly by targeting less active individuals to shift them toward moderate thresholds or enhancing sustained high activity—holds potential to reduce public health expenditures, especially given scarce mental health pharmacotherapy budgets.

We recommend integrating specific validated screening tools—such as the Baecke Inventory used in this study or the International Physical Activity Questionnaire (IPAQ)—into routine primary care consultations to systematically identify insufficiently active patients who would benefit most from intervention, particularly those at higher risk of elevated psychotropic and overall medication expenditures.

## Figures and Tables

**Table 1:** Characteristics of patients of basic health units in the city, 2020 (n = 250).

Variables (N—250)		Less Active	More Active	Total
		*n* (%)	*n* (%)	*n* (%)
	HPA	197 (78.8)	53 (21.2)	250 (100)
Sex	Male	42 (77.8)	12 (22.2)	54 (21.6)
	Female	155 (79.1)	41 (20.9)	196 (78.4)
Residence	BHU Rural	34 (79.1)	9 (20.9)	43 (17.2)
	BHU Urban	163 (78.7)	44 (21.3)	207 (82.8)
Age Range	<50 years	53 (79.1)	14 (20.9)	67 (26.8)
	<60 years	72 (76.6)	22 (23.4)	94 (37.6)
	<70 years	47 (75.8)	15 (24.2)	62 (24.8)
	70 years or more	25 (92.6)	2 (7.4)	27 (10.8)
Occupation	Does not work/no income	65 (83.3)	13 (16.7)	78 (31.2)
	Retired/pensioner	69 (83.1)	14 (16.9)	83 (33.2)
	Formal employment	25 (69.4)	11 (30.6)	36 (14.4)
	Informal/temporary employment	31 (67.4)	15 (32.6)	46 (18.4)
	Retailer	3 (100)	0 (0.0)	3 (1.2)
	Other	4 (100)	0 (0.0)	4 (1.6)
Skin color	White	87 (84.5)	16 (15.5)	103 (41.2)
	Black	13 (72.2)	5 (27.8)	18 (7.2)
	Pardo	79 (77.4)	23 (22.5)	102 (40.8)
	Yellow	1 (50.0)	1 (50.0)	2 (0.8)
	Other race/color	17 (68.0)	8 (32.0)	25 (10.0)
Schooling	Illiterate/EE 1 incomplete	86 (81.9)	19.0 (18.1)	105.0 (42.0)
	EE 1 complete/EE 2 incomplete	54 (78.3)	15 (21.7)	69 (27.6)
	EE 2 complete/SE incomplete	21 (74.4)	8 (27.6)	29 (11.6)
	SE complete/HE incomplete	29 (80.6)	7 (19.4)	36 (14.4)
	HE complete	7 (63.6)	4 (36.4)	11 (4.4)
Socioeconomic Level	Class E	5 (100)	0 (0.0)	5 (2.0)
	Class D	82 (82.8)	17 (17.2)	99 (39.6)
	Class C2	66 (80.5)	16 (19.5)	85 (32.8)
	Class C1	30 (71.4)	12 (28.6)	42 (16.8)
	Class B2	12 (70.6)	5 (29.4)	17 (6.8)
	Class B1	2 (40.0)	3 (60.0)	5 (2.0)
Diagnosis of Depression	Yes	14 (66.7)	7 (33.3)	21 (8.4)
	No	183 (79.9)	46 (20.1)	229 (91.6)
Diagnosis of Anxiety	Yes	13 (76.5)	4 (23.5)	17 (6.8)
	No	184 (79.0)	49 (21.0)	233 (93.2)

Legend: HPA: Habitual Physical Activity; BHU: Basic Health Unit; EE: elementary education; SE: secondary education, HE: higher education. **Source:** Author’s own, prepared using STATA software. **Note:** Author’s own, prepared using STATA software version 16.

**Table 2:** Total spending on medicines and specific spending on psychotropic medicines among the more active and less active patients in APC.

Variables	Medicines					Psychotropic Medicines				
(N—250)	Mean	Diff %	Median	SD	95%CI	Mean	Diff %	Median	SD	95%CI
HPA										
Less active	6.68	32.54	0.75	34.125	1.88; 11.47	0.63	1.61	0.00	4.742	−0.03; 1.30
More active	5.04	ref	0.72	14.105	1.15; 8.93	0.62	Ref	0.00	2.633	−0.10; 1.35
Sex										
Male	5.71	−12.15	0.60	10.692	2.79; 8.63	0.49	−26.87	0.00	2.567	−0.20; 1.19
Female	6.5	ref	0.74	34.538	1.64; 11.37	0.67	Ref	0.00	4.759	0.00; 1.34
Residence										
BHU Rural	4.32	−36.00	0.00	14.823	−0.24; 8.88	0.04	−94.67	0.00	0.177	−0.01; 0.10
BHU Urban	6.75	ref	0.85	33.361	2.18; 11.32	0.75	ref	0.00	4.801	0.10; 1.41
Age range										
<50 years	3.46	−64.29	0.42	7.518	1.65; 5.27	0.24	−7.69	0.00	1.339	−0.08; 0.56
<60 years	3.76	−61.20	0.72	11.718	1.38; 6.14	0.46	76.92	0.00	1.741	0.10; 0.81
<70 years	11.86	22.39	0.78	58.535	−2.78; 26.50	1.48	469.23	0.00	8.383	−0.62; 3.57
70 years or more	9.69	ref	1.82	19.438	2.32; 17.05	0.26	ref	0.00	0.869	−0.06; 0.59
Occupation										
Does not work/no income	3.96	ref	0.75	8.667	2.03; 5.89	0.71	ref	0.00	2.594	0.13; 1.28
Retired/pensioner	10.87	174.49	0.97	51.431	−0.26; 21.99	0.98	38.03	0.00	7.012	−0.53; 2.49
Formal employment	2.11	−46.72	0.00	7.237	−0.26; 4.49	0.03	−95.77	0.00	0.206	−0.03; 0.10
Informal/temporary employment	4.58	15.66	0.77	14.977	0.23; 8.93	0.15	−78.87	0.00	0.648	−0.03; 0.34
Retailer	21.11	433.08	27.62	13.979	5.22; 37.01	0	−100.00	0.00	0	0; 0
Other	5.30	33.84	2.81	6.021	−0.63; 11.23	3.34	370.42	0.00	6.674	−3.23; 9.91
Skin color										
White	9.67	142.36	0.61	46.699	0.54; 18.79	1.25	525	0.00	6.728	−0.06; 2.56
Non-white	3.99	ref	0.75	9.902	2.38; 5.61	0.2	ref	0.00	0.757	0.08; 0.32
Schooling										
Illiterate/EE 1 incomplete	10.05	ref	0.84	46.78	1.06; 19.04	0.93	ref	0.00	6.315	−0.29; 2.14
EE 1 complete/EE 2 incomplete	3.91	−61.09	1.03	7.56	2.09; 5.72	0.50	−46.24	0.00	1.908	0.04; 0.95
EE 2 complete/SE incomplete	3.94	−60.8	0.45	7.81	1.08; 6.79	0.68	−26.88	0.00	3.43	−0.57; 1.94
SE complete/HE incomplete	2.57	−74.43	0.16	5.89	0.64; 4.51	0.17	−81.72	0.00	0.78	−0.08; 0.43
HE complete	4.61	−54.13	0.00	12.79	−2.98; 12.21	0.03	−96.77	0.00	0.116	−0.03; 0.10
Socioeconomic Level										
Class E	1.93	−78.44	1.00	1.965	0.20; 3.66	0.20	Ref	0.00	0.445	−0.19; 0.59
Class D	5.21	−41.79	0.84	13.075	2.63; 7.80	0.46	130	0.00	1.683	0.13; 0.79
Class C2	2.97	−66.82	0.72	6.521	1.55; 4.38	0.27	35	0.00	1.351	−0.02; 0.57
Class C1	17.47	95.2	0.47	71.559	−4.28; 39.22	2.09	945	0.00	10.156	−1.00; 5.18
Class B2	2.06	−76.98	0.00	5.015	−0.33; 4.46	0.07	−65	0.00	0.279	−0.07; 0.20
Class B1	8.95	ref	0.00	18.949	−7.74; 25.64	0.00	−100	0.00	0.00	0.00; 0.00
Diagnosis of Depression										
Yes	34.07	798.94	4.76	9.551	2.54; 5.03	5.93	3853.33	2.16	1.146	−0.00; 0.29
No	3.79	ref	0.46	100.064	−11.47; 79.62	0.15	ref	0.00	13.828	−0.36; 12.22
Diagnosis of Anxiety										
Yes	33.71	678.52	6.87	110	−22.85; 90.26	6.19	2591.3	1.00	15.36	−1.70; 14.09
No	4.33	ref	0.58	11.64	2.83; 5.83	0.23	ref	0.00	1.36	0.05; 0.40

**Legend:** 95%CI: Confidence interval at 95% level; SD: Standard deviation; HPA: Habitual Physical Activity; BHU: Basic Health Unit; Diff %: mean difference in %; ref: reference. **Source:** Author’s own, prepared using STATA software version 16. **Legend:** EE: elementary education; SE: secondary education, HE: higher education; ref: reference.

**Table 3:** Mean effect of HPA on total spending on medicines and specific spending on psychotropic medicines in patients of basic health units, 2020 (n = 250).

	Spending on Medicines				Spending on Psychotropic Medicines			
Model	Coefficient	Statistic	Standard Error	Standard Error	Coefficient	Statistic	Standard Error	Standard Error
				Boostrap				Boostrap
NN(1)	−34.83 ***	**−2.86 ^t^**	**12.177**	**15.346**	−4.34 ***	**−2.57 ^t^**	**1.689**	**2.032**
NN(5)	−15.52 ***	−2.71 ^t^	5.732	10.224	−1.80 **	−2.21 ^t^	0.814	1.324
Kernel	−19.32 ***	−4.53 ^t^	4.263	12.052	−2.53 ***	−3.95 ^t^	0.641	1.64
Radius	−22.33 ***	−4.63 ^t^	4.823	9.914	−2.86 ***	−3.99 ^t^	0.718	1.25
IPW	−30.03 **	−2.10 ^z^	14.293	-	−3.74 *	−1.94 ^z^	1.93	-
IPWRA	−32.43 **	−2.12 ^z^	15.277	-	−4.02 **	−1.98 ^z^	2.034	-

**Source:** produced by the authors. Legend: NN = Nearest Neighbor. **Note1:** NN(1) with replacement; NN(5) with replacement; Radius with 0.1% caliper and common support; Kernel with window value of 0.06 and common support. PSM: standard errors generated by bootstrap reps(50). IPW and IPWRA with robust standard errors generated by Stata’s effects command and with 200 bootstrap replications. All models used the covariates from Table 2. **Note2:** NN(1) (nearest neighbor with one match) exhibits larger effect sizes than other algorithms due to its restriction to the single most similar control unit, minimizing bias from imperfect matches but increasing variance through reduced effective sample size in propensity score matching analyses. Common support satisfied. Standard error of PSM estimated with 50 bootstrap replications. Standard error of IPW and IPWRA models estimated with 200 bootstrap replications. *** *p* < 0.01, ** *p* < 0.05, * *p* < 0.1 indicate the level of statistical significance. ^t^ t-statistic. ^z^ z-statistic.

**Table 4:** Difference in means of observable characteristics of the more and less active groups, before and after matching.

Variables	Before Matching			After Matching		
	Less Active	More Active	*p*-Value	Less Active	More Active	*p*-Value
BHU Urban	0.827	0.83	0.957	0.924	0.83	0.141
Female	0.789	0.773	0.808	0.773	0.773	1
<60 years	0.362	0.415	0.485	0.396	0.415	0.845
<70 years	0.254	0.283	0.673	0.301	0.189	0.257
70 years or more	0.13	0.038	0.059	0.057	0.038	0.651
Retired/pensioner	0.357	0.264	0.21	0.207	0.264	0.497
Formal employment	0.207	135	0.196	0.188	0.207	0.81
Informal/temporary employment	0.167	0.283	0.061	0.321	0.283	0.676
Retailer	0	0				
Other	0	0				
Black	0.07	0.094	0.561	0.094	0.094	1
Pardo	0.405	0.434	0.711	0.509	0.434	0.441
Yellow	0.005	0.019	0.346	0.094	0.019	0.094
Other race/color	0.086	0.151	0.171	0.132	0.15	0.783
EE 1 complete/EE 2 incomplete	0.265	0.283	0.794	0.226	0.283	0.508
EE 2 complete/SE incomplete	0.097	0.151	0.272	0.17	0.151	0.794
SE complete/HE incomplete	0.151	0.132	0.728	0.113	0.132	0.77
HE complete	0.038	0.075	0.252	0.038	0.075	0.405
Class D	0.438	0.32	0.128	0.472	0.32	0.114
Class C2	0.329	0.301	0.704	0.245	0.301	0.518
Class C1	0.157	0.226	0.238	0.151	0.226	0.325
Class B2	0.065	0.094	0.465	0.094	0.094	1
Class B1	0.011	0.057	0.041	0.038	0.057	0.651
Diagnosis of Depression	0.07	0.132	0.154	0.094	0.132	0.544

**Legend:** BHU: Basic Health Unit; **Note:** Comparison of means before and after matching, *t* test. **Source:** produced by the authors. **Legend:** EE: elementary education; SE: secondary education, HE: higher education **Note:** Comparison of means before and after matching, *t* test. **Source:** produced by the authors.

**Table 5:** Results of placebo regressions for PSM.

Placebo Variables			
	BMI	Spending on Dental Consultations	Frequency of Dental Consultations
Being more active	0.841	−2.47	−0.271
Standard error	1.288	2.275	0.294
Observations	250	250	250
t statistic	0.65	−1.08	−0.92

**Legend:** BMI: Body Mass Index. **Note:** Generated based on the full model and nearest neighbor matching with replacement.

## Data Availability

Some or all data and models that support the findings of this study are available from the corresponding author upon reasonable request.

## Abbreviations

The following abbreviations are used in this manuscript:

PSM	Propensity Score Matching
IPW	Inverse Probability Weighting
IPWRA	Inverse Probability Weighted Regression Adjustment
HPA	Habitual Physical Activity
